# Shortened leukocyte telomere length in young adults who use methamphetamine

**DOI:** 10.1038/s41398-021-01640-z

**Published:** 2021-10-09

**Authors:** Yen-Feng Lin, Po-Yu Chen, Hsing-Cheng Liu, Yi-Lung Chen, Wei-Hern Chou, Ming-Chyi Huang

**Affiliations:** 1 Center for Neuropsychiatric Research, National Health Research Institutes, 35 Keyan Road, 350 Zhunan, Miaoli County Taiwan; 2 School of Medicine, National Yang-Ming University, No.155, Sec. 2, Linong St., 112 Taipei City, Beitou District Taiwan; 3 Department of Psychiatry, Taipei City Psychiatric Center, Taipei City Hospital, 309, Songde Road, 110 Taipei City, Xin-Yi District Taiwan; 4 Department of Psychiatry, School of Medicine, College of Medicine, Taipei Medical University, 250 Wu-Xing St., 110 Taipei, Taiwan; 5 Psychiatric Research Center, Taipei Medical University Hospital, 250 Wu-Hsing Street, 110 Taipei, Taiwan

**Keywords:** Prognostic markers, Addiction, Physiology

## Abstract

Methamphetamine (METH) use, most prevalent in young adults, has been associated with high rates of morbidity and mortality. The relationship between METH use and accelerated biological aging, which can be measured using leukocyte telomere length (LTL), remains unclear. We examined whether young adult METH users have shorter LTL and explored the relationship between characteristics of METH use and LTL by using Mendelian randomization (MR) analysis. We compared the LTL for 187 METH users and 159 healthy individuals aged between 25 and 34 years and examined the relationship of LTL with METH use variables (onset age, duration, and maximum frequency of METH use) by using regression analyses. In addition, 2-stage-least-squares (2SLS) MR was also performed to possibly avoid uncontrolled confounding between characteristics of METH use and LTL. We found METH users had significantly shorter LTL compared to controls. Multivariate regression analysis showed METH use was negatively associated with LTL (β = −0.36, *P* < .001). Among METH users, duration of METH use was negatively associated with LTL after adjustment (β = −0.002, *P* = .01). We identified a single nucleotide polymorphism (SNP) rs6585206 genome-wide associated with duration of METH use. This SNP was used as an instrumental variable to avoid uncontrolled confounding for the relationship between the use duration and LTL shortening. In conclusion, we show that young adult METH users may have shorter LTL compared with controls and longer duration of METH use was significantly associated with telomere shortening. These observations suggest that METH use may accelerate biological senescence.

## Introduction

Illicit methamphetamine (METH) use leads to considerable public health, legal, and social problems worldwide and contributes substantially to global disease burden, causing a 37% increase in the disability adjusted life years over the past decades [1]. The burden of diseases is mainly derived from a number of adverse physical and mental health consequences associated with METH use [2, 3]. Individuals who use METH have poorer physical health [4] and carry an elevated risk (~3–6 times) of mortality compared to general population [5, 6]. The mortality risk is even greater than users with cannabis, cocaine, or alcohol [7]. METH use is most prevalent in young adults, particularly those aged 25–34 years [8], and this raises grave concerns because this population carries greater risk for long-term negative outcomes [9].

The underlying mechanisms through which METH increase a higher risk of morbidity and mortality remains unknown. METH use causes systemic toxicity, affecting immune function [10] and impairing the function of multiple organs [2]. A variety of medical complications have been related to METH use, such as tooth decay (‘meth mouth’) [11], pulmonary hypertension [12], hepatotoxicity [13], cardiovascular diseases and strokes [14, 15], and blood-borne and sexually transmitted infections [16]. These complications may indicate physiological characteristics of premature aging process in METH users. In line with it, METH use has been associated with an increased risk of developing age-related illnesses such as Parkinson’s disease [17, 18]. Preclinical studies also have indicated that METH may accelerate cellular senescence, i.e., when the aging at the cellular level exceeds that of which is expected by chronological age, and increase the speed of functional decline of organisms [19, 20].

In recent years, the relationship between psychiatric disorders and accelerated biological aging has gained much attention [21]. Telomeres are protective caps at the end of chromosomes composed of base pairs of TTAGGG repeats [22], which preserve genomic integrity and stability by protecting against DNA damage [23] The telomeres length declines with each cell division and when telomeres reach a critically minimal length the cell undergoes cellular senescence or apoptosis [24]. In adults, shortened leukocyte telomere length (LTL) is widely considered as a biomarker of biological aging [25, 26]. Shorter LTL has been associated with aging-related diseases such as atherosclerotic cardiovascular diseases [27] as well as increased risks for morbidity and mortality [28]. These observations suggest a link between LTL shortening and METH use. So far the role of shortened LTL in drug abuse has been less studied [29].

Yang et al. reported that drug abusers overall had reduced LTL, but when they classified the drugs of abuse into depressant drugs, stimulant drugs, and hallucinogenic drugs they found those taking stimulant drugs (such as METH and methylenedioxymethamphetamine [MDMA]) did not exhibit shorter LTLs [30]. In contrast, reports in cocaine users demonstrated that cocaine use promotes the shortening of telomere length [31, 32]. Furthermore, cocaine abusers reporting a history of childhood abuse and neglect displayed significantly shorter telomeres than those without such history [32], suggesting adverse childhood experiences (ACEs) might contribute to the shortening of LTL in drug abusers.

Given that we are not aware of studies that had examined the relationship between METH use and LTL and in order to test our hypothesis that METH use will exhibit accelerated cellular senescence as measured by LTL, in this study we first compared the LTLs of young adult METH users and healthy controls. Second, we investigated the associations of LTL with the clinical characteristics, such as ACEs or METH use variables (e.g., age of first use and duration and frequency of METH use) in METH group. Third, because observational studies are limited by the inability to completely measure and adjust for confounders that are shared between exposure and outcomes, the true exposure–outcome relationship may be obscured [24]. Mendelian randomization (MR) has been suggested as a potential solution to this problem; in this approach, exposure-associated genetic variants are employed as instrumental variables (IVs) to reduce the influence of confounding on an observed association [25, 33]. In the present study, we identified single nucleotide polymorphisms (SNPs) that were associated genome-wide with the METH use variables assessed in this study and performed MR to possibly avoid uncontrolled confounding between characteristics of METH use and LTL in young adults.

## Material and methods

This cross-sectional study was performed at Taipei City Psychiatric Center (TCPC), Taipei Cty Hospital, in accordance with the Declaration of Helsinki and was approved by the hospital’s institutional review board (IRB No: TCHIRB-10803012). Written informed consent was obtained from all participants after we described the study comprehensively.

### Participants

We recruited METH users from the Department of Addiction Sciences of TCPC. Those who met the following inclusion criteria were invited to participate in this study: (1) age between 25 and 34 years; (2) using METH as their main substance of abuse in the past and at least once per month in the preceding year validated by urine toxicology screening for METH as well as subjective and at least one family member’s or close friend’s report; (3) receiving a Diagnostic and Statistical Manual of Mental Disorder, 5th Edition diagnosis of METH use disorder, as verified by 2 board-certified psychiatrists; and (4) ability to read Chinese and provide informed consent. Individuals were excluded from participating if they (1) had ever used another substance (except nicotine) with a frequency more than once per month; (2) had a systemic medical illness such as a sexually transmitted disease (e.g., human immunodeficiency virus or syphilis), acute or chronic liver diseases (eg, hepatitis B and C), or inflammatory or rheumatological diseases; (3) had a history of major psychiatric disorders (including schizophrenia, bipolar disorder, and major depressive disorder) or psychotropic drug treatment (including antipsychotics, mood stabilizers, or antidepressants) except sedatives or hypnotics; and (4) had systemic or neurological diseases such as atherosclerotic coronary artery diseases or heart failure, respiratory diseases, diabetes mellitus, chronic kidney disease, or recent infections or had used medications chronically to treat other physical illnesses.

A healthy control group was enrolled from the Health Examination Center of Taipei City Hospital, Jen-Ai Branch, Taipei, Taiwan. The control participants had to meet the following inclusion criteria: (1) no known physical or psychiatric illnesses as described earlier according to clinical interviews and routine laboratory tests; (2) not meeting the diagnostic criteria for any substance (except nicotine) use disorder in the past; and (3) negative results of blood alcohol concentration and urine toxicology tests.

### Clinical assessment

METH users underwent a structured interview face-to-face by a trained psychologist using the Chinese version of the Diagnostic Interview for Genetic Studies [34] to assess demographic information, medical and psychiatric illnesses, METH use variables (including age of the first METH use, duration of METH use, and frequency of METH use), and history of polysubstance abuse, which was defined in this study as having used other substance apart from METH and nicotine but with a frequency less than once per month. Smoking history information was determined by self-reported smoking status (never vs. ever smoker) and pack-years (average number of packs of cigarettes they smoked each day multiplied by the total number of years they have smoked) [35]. Because adverse childhood experiences (ACEs) have been previously associated with decreased telomere length in people with drug addiction [32, 36], the self-administered ACEs questionnaire adapted from the Family Health History Questionnaire [37] was also collected. Information on ACEs was obtained using questions with binary responses regarding exposure to the following nine types of experience during the participants’ first 18 years of life: T emotional, physical, and sexual abuse; having a battered mother; having parents who separated or divorced; having household members who were incarcerated; being exposed to problematic substance use in the household; being exposed to mental illness in the household; and having a disadvantaged socioeconomic status. The total number of ACE categories for each participant was recorded as his or her ACE score (range, 0–9).

### Leukocyte telomere length

LTL was measured using genomic DNA, which was extracted from peripheral blood leukocytes collected into tubes containing ethylenediaminetetraacetic acid (EDTA) (BD Vacutainer, NJ, USA) by using the Easy Blood Genomic DNA Purification Kit (GMbiolab, TCC, Taiwan) and stored at −80 °C. DNA quality and concentration was measured using the Nanodrop 2000 (Thermoscientific, DE, USA). In addition, to ensure quality control (QC), DNA integrity was affirmed before measuring LTL by using the Southern blot analysis method.

To assess the relative LTLs of our participants, we performed two separate quantitative polymerase chain reactions (qPCRs) using the StepOnePlus™ Real-Time PCR System (Applied Biosystems, CA, USA) with the StepOne software v2.3 used to generate the outputs. In the first qPCR reaction, we examined the telomere repeat region (TTAGGG). In the second reaction, we assayed a single copy gene *HBG4* (β-globin), which we used as an internal control to correct for minor differences in DNA concentration across samples [38]. The primer sequences were as follows: telomere forward primer: 5’ GGTTTTTGAGGGTGAGGGTGAGGGTGAGGGTGAGGGT; telomere reverse primer: TCCCGACTATCCCTATCCCTATCCCTATCCCTATCCCTA; HBG3 (β-globin) forward primer: TGTGCTGGCCCATCACTTTG; and HBG4 reverse primer: ACCAGCCACCACTTTCTGATAGG. Each PCR reaction involved 50 ng of genomic DNA, 1X KAPA SYBR® FAST qPCR Master Mix (KAPA Biosystems, MA, USA), 200 nM telomere primer/200 nM HBG3 primer, and ultrapure water, making up a final volume of 20 μL. The thermal cycling profile began with enzyme activation at 95 °C for 10 min, followed by 40 cycles of 95 °C for 15 s and 60 °C for 60 s for telomere and β-globin, respectively. To detect any DNA contamination, each plate contained two negative controls (ultrapure water). The telomere/single copy gene (HBG4) (T/S) ratios were calculated using the 2^−ΔCt^ method, where ΔCt = average Ct_Tel_ − average Ct_HBG4_. Because each experimental sample was assayed in duplicate, 2 T/S ratio results were obtained for each sample; the final reported result for a sample in a given run is the average of the 2 T/S values.

### Genotyping and imputation

Genome-wide genotyping for individuals with METH use was performed using the Axiom Genome-Wide TWB 2.0 Array Plate with 686,463 SNPs. We excluded individuals with dish QC < 0.82, QC call rate < 97%, high heterozygosity rate, genotypic and phenotypic sex mismatch, and closely related individuals. For QC of SNPs, we excluded SNPs with missing genotype rate > 0.05, deviation of Hardy–Weinberg equilibrium as *P* < 7.306 × 10^−8^, minor allele frequency < 0.01, and genotyping not in ATCG. Genotyping data were imputed using Minimac4 on the Michigan Imputation Server, with reference-panel-extracted Eastern Asian ancestry from 1000 Genomes Phase 3 version 5. After imputation, we further filtered the imputed and genotyped variants to include only SNPs with minor allele frequency ≥ 0.01 and with imputation information score ≥ 0.8.

### Statistical analysis

#### Association between LTL and METH use

We used univariate and multivariate linear regressions to assess the association between LTL and METH use. The multivariate model was adjusted for the potential confounders age, sex, marital status, educational attainment, smoking, and total ACE score. Among METH users, in order to evaluate the association of LTL with METH use variables, we used univariate and multivariate linear regressions with the multivariate regressions adjusting for age, sex, marital status, educational attainment, cumulative smoking dose (defined as packs of tobacco smoked per day multiplied by smoking years), ACE scores, and METH use variables. We also performed multivariate linear regression analyses stratified by having a history of polysubstance use and urine toxicology result for METH as a sensitivity analysis to examine whether the associations that were observed in the entire sample of METH users still held in the subgroups.

#### Genome-wide association analysis of METH use phenotypes and MR

For each METH use variable discovered to be associated with LTL, we performed MR to avoid potentially uncontrolled confounding rather than simply reporting associations between METH use and LTL. We performed genome-wide association analyses with 5,374,651 imputed and genotyped variants by using PLINK 1.9, and then we selected significant SNPs as IVs to estimate the effect of METH use variables on LTL by using 2-stage least-squares (2SLS) MR [39], executed using R software. The first stage of 2SLS was a regression of exposure (i.e., METH use variable) on the SNP instruments. The second stage was a regression of LTL on the fitted exposure value predicted by the stage-1 model. The effect of METH use variable on LTL could be estimated by the regression coefficient for the change in outcome due to the unit change in exposure in the second model (see Supplementary Methods). To adjust for population stratification in genome-wide association analyses, we included the 10 principal components as covariates in the linear regression model.

## Results

### Association of LTL shortening with METH use in the entire sample

We enrolled a total of 187 individuals who used METH (METH group) and 159 healthy controls (control group). The groups’ demographic and clinical characteristics are presented in Table 1. No differences were observed between the two groups in age, sex, or marital status. The METH users had lower educational attainment (*P* < .0001), were more likely to be smokers, and reported more ACEs (*P* < .0001) than the control group. Notably, LTL was significantly shorter in the METH group relative to the control group (*P* < .0001).

**Table 1:** Demographic and clinical characteristics for methamphetamine (METH) users and healthy controls.

	METH users	Healthy controls	*P*-value
	(*N* = 187)	(*N* = 159)	
Age (years), mean (SD)	29.52 (2.89)	28.99 (2.89)	0.09^b^
Sex			
Female, *N (%)*	27 (14.4)	33 (20.8)	0.12^c^
Marital status			
Married, *N (%)*	25 (13.6)	30 (19.1)	0.17^c^
Education (years), median (Q1, Q3)	10 (9, 12)	16 (16, 16)	<0.0001^d^
Smoking			
Ever smoker, *N (%)*	160 (94.7)	36 (22.6)	<0.0001^c^
Cumulative smoking dose (pack-years), median (Q1, Q3)	11 (5, 20)		
ACEs score^a^, median (Q1, Q3)	2 (1, 3)	1 (0, 2)	<0.0001^d^
METH use variables			
Onset age of METH use (years), median (Q1, Q3)	23 (18, 28)		
Duration of METH use (months), median (Q1, Q3)	10 (3, 59)		
Maximum frequency of METH use (times/month), median (Q1, Q3)	12 (4, 30)		
Polysubstance use, *N (%)*	68 (36.4)		
Relative T/S ratio, median (Q1, Q3)	0.70 (0.31, 1.06)	1.01 (0.63, 2.04)	<0.0001^d^

^a^ACE score: the summed number of ACEs categories for each participant (range, 0–9).

^b^Two-sample *t*-test.

^c^Chi-square test.

^d^Mann–Whitney rank test.

Because the distribution of LTL measures was positively skewed, we employed a square root transformation (see Supplementary Fig. S1) and used the transformed LTL as the dependent variable in the linear regression analyses. The results of these analyses for the associations of LTL with METH use and demographic and clinical characteristics are presented in Table 2. We observed that METH use was associated with LTL shortening (unadjusted *P* < .001), and this result remained significant after multivariable adjustment (adjusted *P* < .001). On average, METH users had a 0.29-unit reduction in LTL measure (i.e., the square root of relative T/S ratio) than nonusers, adjusting for potential confounders.

**Table 2:** Univariate and multivariate linear regression to examine the association of telomere length (dependent variable) with demographic, clinical, and METH use variables (independent variables) for the entire sample (*N* = 346).

Independent variable	Dependent variable: square root of the relative T/S ratio			
	Univariate analysis		Multivariate analysis^b^	
	Beta	95% CI	Beta	95% CI
Age	−0.01	−0.02, 0.01	−0.01	−0.32, 0.002
Sex				
Female	−0.08	−0.20, 0.04	−0.09	−0.22, 0.03
Marital Status				
Married	0.12	−0.01, 0.24	**0.13**	**0.002, 0.26**
Education years	**0.03**	**0.02, 0.05**	−0.01	−0.04, 0.01
Ever smoking	**−0.19**	**−0.28, −0.10**	0.04	−0.11, 0.18
ACEs score^a^	**−0.04**	**−0.07, −0.02**	−0.01	−0.04, 0.02
METH use	**−0.29**	**−0.38, −0.21**	**−0.36**	**−0.54, −0.18**

Bold font indicates statistical significance (*p* < 0.05).

*95% CI* 95% confidence interval.

^a^ACE score: the summed number of ACEs categories for each participant (range, 0–9).

^b^Multivariate analysis adjusted for age, sex, marital status, educational attainment, ever smoking, and total ACEs score.

### Associations between LTL and METH use variables in the METH group

Table 3 presents the results of linear regressions for the associations between LTL and the demographic and clinical characteristics of the METH group. We observed that duration of use was significantly associated with LTL shortening (unadjusted *P* = .04; adjusted *P* = .01), suggesting that a cumulative decrease in LTL might occur along with increasing exposure to METH. For every 1 month increase in the duration of METH use, the LTL measure (i.e., the square root of relative T/S ratio) reduced by 0.002 unit averagely, adjusting for potential confounders. The association was not observed for onset age or frequency of METH use. In subgroup analyses, we found the association between duration of METH use and LTL was significant in the subgroups of METH users without a history of polysubstance abuse (i.e., METH-only users) (see Supplementary Tables S1) and those with positive urine METH test (see Supplementary Tables S2).

**Table 3:** Univariate and multivariate linear regression to examine the association of telomere length (dependent variable) with demographic, clinical, and METH use variables (independent variables) for METH users (*N* = 187).

Independent variable	Dependent variable: square root of the relative T/S ratio			
	Univariate analysis		Multivariate analysis^b^	
	Beta	95% CI	Beta	95% CI
Age	−0.001	−0.019, 0.016	−0.007	−0.037, 0.024
Sex				
Female	**−0.213**	**−0.354, −0.072**	−0.125	−0.364, 0.114
Marital status				
Married	0.148	−0.0003, 0.296	**0.254**	**0.041, 0.467**
Education years	0.007	−0.019. 0.034	−0.002	−0.046, 0.042
Smoking				
Cumulative smoking dose (pack-years)	0.002	−0.004, 0.007	0.003	−0.004, 0.009
ACEs score^a^	−0.019	−0.046, 0.009	−0.018	−0.058, 0.022
METH use variable				
Onset age of METH use	0.005	−0.004, 0.014	−0.008	−0.025, 0.008
Duration of METH use	**−0.001**	**−0.002, −0.00005**	**−0.002**	**−0.004, −0.0001**
Maximum frequency of METH use	−0.0004	−0.002, 0.0008	−0.0002	−0.002, 0.001
Polysubstance use	−0.008	−0.113, 0.098	0.109	−0.064, 0.281

Bold font indicates statistical significance (*p* < 0.05).

*95% CI* 95% confidence interval.

^a^ACE score: the summed number of ACEs categories for each participant (range, 0–9).

^b^Multivariate analysis adjusted for age, sex, marital status, educational attainment, total ACEs score, cumulative smoking dose, polysubstance use, and METH use variables.

### MR to avoid uncontrolled confounding between METH use duration and LTL

As stated, METH use duration was significantly associated with LTL shortening. We further performed 2SLS MR to possibly reduce the impact of uncontrolled confounding for the relationship between the duration of METH use and LTL. In stage 1, we performed genome-wide association analysis of METH use duration and identified a significantly associated SNP rs6585206 on chromosome 10 (*P* = 8.76E-13) (see Supplementary Fig. S2). In stage 2, we used rs6585206 as the IV and obtained a significant estimated effect of duration of METH use on LTL (beta = −0.002 [95% CI: −0.004, −0.0005]; *t* = −2.50; *P* = .01). The result of MR analysis suggested that every 1 month increase in the duration of METH use reduced the LTL measure by 0.002 unit averagely. The effect of METH use duration on LTL estimated by MR was very close to that obtained from our multivariate regression analysis, suggesting confounding might have been well-controlled by considering many covariates.

## Discussion

In the present study, we observed that young adults who used METH had shorter mean telomere length than healthy individuals after adjustment for potential confounders including ACEs. Among the METH group, we observed the duration of METH use was significantly correlated with a reduced LTL, suggesting that longer exposure to METH may shorten telomeres. In addition, the MR analysis in which the SNP rs6585206, which was significantly associated with METH use duration based on genome-wide association data, was used as the IV to further avoid uncontrolled confounding for the observed effect of duration of METH use on the risk of LTL shortening.

LTL shortening has been noted previously in the context of substance. For example, indivudals with cocaine use [31, 32], alcohol abuse [40], in particular binge drinking [41], and heroin abuse [42] have been reported to have significantly shorter LTL. Yang et al., in an examination of the LTL in individuals recruited from drug rehabilitation centers, found that drug abusers generally had reduced LTL than controls; however, no significant association was observed between LTL and stimulant drugs or METH use [30]. In addition, different age groups, from 15 to 61 years, were included [30], and because age is an essential modifier of LTL decline, the results did not enable an estimation of LTL change in a young adult population. Our results fill the knowledge gap by providing evidence that young people who use METH have significantly shortened telomeres. Similarly, some evidence has been obtained that cocaine use promotes telomere shortening [31, 32]. Although only a few studies have addressed the relevance of telomere shortening in people with substance use disorders, an early study demonstrated that the common physical biomarkers of aging—such as C-reactive protein, lymphocyte count, alanine aminotransferase, creatinine, urea, and insulin-like growth factor-1 are altered in drug abusers [43]. Because LTL shortening is considered to be a biomarker of accelerated aging, this result also lends support to our findings of an association between the disrupted aging process and METH use. In addition, one animal study revealed that rats administered METH displayed impairments similar to aged rats in dopamine, serotonin, and metabolite levels in multiple areas of the brain (the striatum, prefrontal cortex, and hippocampus) and therefore suggested that METH can potentially be used to mimic age-related effects on neurochemical systems [20]. These observations collectively suggest that METH use may lead to premature aging process, thereby accelerating the speed of physiological function decline in organisms [19, 20] and causing various disorders.

The mechanisms underlying the relationship between METH use and LTL shortening are unclear. Accumulating evidence suggests that telomere length is a result of the combined effect of oxidative stress, inflammation, and repeated cell replication, thus creating associations between a decline in telomere length and aging and age-related diseases. While the immune systme dysregultion or abnormal inflammatory response may promote the progresson of cellular senescence secondary to METH [44, 45], most of the evidence obtained in past decades indicates that increased levels of oxidative stress play a critical role in the aging process [46]. Oxidative stress is defined as the cytotoxic consequence of an imbalance between reactive oxygen species (ROS) production and antioxidant defenses. Excess ROS result in damage to macromolecules including DNA, thereby causing breaks in DNA strands. This rate of damage increases with advancing age and is further increased by disease conditions when antioxidant and repair mechanisms are compromised. Because guanine is the most easily oxidized of the natural bases and TTAGGG repeats of telomeres are the preferred sites for conversion of G to 8-oxoG in vitro [47], telomeres are particularly susceptible to oxidative-damage-mediated attrition [48], We previously noted that METH abusers, compared with healthy controls, are subjected to higher systemic oxidative stress and have inadequate antioxidant defenses, with the oxidative stress persisting after early abstinence [49]. Furthermore, the level of the oxidative DNA adduct 8-hydroxy-2’-deoxyguanosine (8-OHdG), which reflects ROS-induced DNA damage in the body, was significantly elevated without improvement following METH discontinuation [50]. Therefore, although we did not measure the oxidative stress indices in this study, it is possible that increased oxidative damage contributes to, at least in part, the telomere shortening in METH abusers. In addition, the observation of a persistent increase in oxidative stress [49, 50] implies accumulation of DNA damage over time in METH abusers. Furthermore, the proinflammatory cytokines that are increased in response to prolonged oxidative stress or antioxidant reduction lead to enhanced shortening of telomeres [51]. These observations collectively lend support to the finding of the present study that longer duration of METH exposure is associated with shorter LTL. Future longitudinal studies that follow the telomere length and erosion while including measures of oxidative stress may shed light on the pathophysiological processes or mechanistic links responsible for premature mortality in METH abusers.

Telomere shortening and telomere stability is also regulated by the enzyme telomerase. Telomerase is a cellular reverse transcriptase that can recognize the telomeric DNA repeat sequence and synthesize a new copy of the repeat, thereby elongating the telomere and reducing telomere loss [51]. In an early report by Cheng et al. [42], both telomere length and telomerase activity in drug users were observed to be significantly decreased compared with those in controls [42]. In this study, although we did not measure telomerase activity, which may counteract telomere attrition in leukocytes [52], based on the observations of Cheng et al., drug abuse may be associated with an accelerated aging process involving altered telomerase activity, which will further influence the LTL.

The study has several limitations. First, because this was cross-sectional, the possibility of reverse causation may have led to misinterpretation of the observed association between METH abuse and LTL, i.e., LTL shortening might precede and cause the individuals to use METH. Second, although we have considered potential confounders, unmeasured confounding remains a concern because we obtained insufficient information about many known aging-related factors, such as physical activity [53], dietary pattern or antioxidant intake [54], hormones [55], omega-3 fatty acids [56], sleep [57], use of benzodiazepines [30], and chronic viral infections (e.g., Cytomegalovirus or Epstein Barr virus) [58]. Third, although our MR approach may further alleviate the potential bias introduced by unmeasured or poorly controlled confounding, a causal interpretation should be treated with caution. SNP rs6585206, that we used as the IV, was located in *TCF7L2* gene, which has been reported to be associated with schizophrennia [59] and type 2 diabetes [60]. Since shortened LTL was also observed in patients with schizophrenia [61] and diabetes [62], a pleiotropic association of the SNP or linkage disequilibrium with other genetic variants that are also related to LTL could potentially violate the assumption that the IV is only related to the outcome via its effect on the exposure [63, 64]. Fourth, we measured telomere length only in leukocytes in peripheral blood. However, because peripheral leukocytes exhibit a rate of age-dependent telomere attrition similar to that of other somatic tissues and telomere length is strongly correlated between tissues, and the length of telomeres are strongly correlated between tissues [65], using leukocytes for measuring telomere length should be satisfactory and have ensured high validity of our results. In addition, although PCR amplification of telomere repeats relative to a single copy gene is not a direct measure of a telomeric region, it is a widely used method that can provide results strongly correlated with those of the gold-standard Southern blot based on average LTL [66]. Finally, we enrolled a sample of METH users who had no history of major systemic or psychiatric diseases. Therefore, our results could not be generalized to those with physical or psychiatric comorbidities that are common in METH users. Also, we lacked the information of depressive and anxiety symptoms and thus were unable to evaluate their impact on the study results.

Despite these limitations, to our best knowledge, this study is the first to evaluate the potential role of METH use in the risk of premature aging in young adults. We provide novel evidence that METH users have decreased LTL and that longer exposure to METH is associated with a shorter LTL. The MR analysis further reduce possible influence of uncontrolled confounding for the dose-dependent relationship between METH use duration and LTL. Because LTL has been previously considered a marker of cellular aging, our results indicate that METH use may accelerate the biological senescence of the human body even in young people. Future studies are required to determine whether telomere shortening can be mitigated over time when users quite METH and to identify the mechanisms that underlie the association. Prompt identification and treatment of patients with METH use disorder may help reduce the speed of telomere shortening and susceptibility to age-related illnesses.

## Supplementary information

Supplementary Methods and Supplementary Tables
