# Supplementary material for: Shortened leukocyte telomere length in young adults who use methamphetamine
Source: Transl Psychiatry. 2021 Oct 9;11:519. doi: 10.1038/s41398-021-01640-z (PMC8502172; doi:10.1038/s41398-021-01640-z)

**Shortened Leukocyte Telomere Length in Young Adults Who Use Methamphetamine**

**SUPPPLEMENTARY MATERILS**

**Supplementary Methods**

**Mendelian randomization**

A Mendelian randomization (MR) study design uses genetic variants as instrumental variables (IVs) for exposure to investigate the causal relationship between exposure and outcome. IVs are commonly used to minimize the potential confounding bias in observational studies. The following directed acyclic graph presents the causal relationship between genetic variants (G), exposure (E), outcome (Y), and confounding factors (U).

G

E

Y

U

β_causal_

γ

To investigate the effects of E on Y (β_causal_), adjusting for U to obtain an unbiased estimate for β_causal_ is needed in observation studies. However, it is difficult to directly adjust either unmeasured or unknown confounding factors. MR may be a possible solution to this issue, using the effect of G on E and the effect of G on Y to estimate the average effect of E on Y.

There are three essential definitions for an IV. First, G has a causal effect on E (γ). Next, G affects Y only through E; that is, there is neither direct effect of G on Y nor the effect of G on Y through other components. Third, G does not share common causes with Y; specifically, there is no direct effect of U on G. As a result, genetic variants would be suitable to be instrumental variables (IVs) in genetic studies if there is no pleiotropic effect.

**Two-stage least squares**

A common approach to implement MR with individual-level data in a single sample is two-stage least squares (2SLS). The first stage is a standard GWAS, where regression analysis for each SNP is performed to examine its association with the exposure (E) to identify potential instrument variables (G). The exposure (E) is regressed on the genetic instrument variable(s), which either be a single SNP, multiple SNPs, or a risk score summing risk allele count based on multiple SNPs. The predicted values of the exposure are taken from the first-stage regression model. In the second stage, the exposure estimate is predicted from the model in the first stage and regressed on the outcome with statistical adjustment of the standard errors. The effect of E on Y ($\beta_{causal}$) is then estimated and tested for significance. The β-coefficient from the second stage can be interpreted as the change in the outcome for linear regression (or the log odds ratio of disease for logistic regression) per unit increase in the exposure due to the genetic instrument.


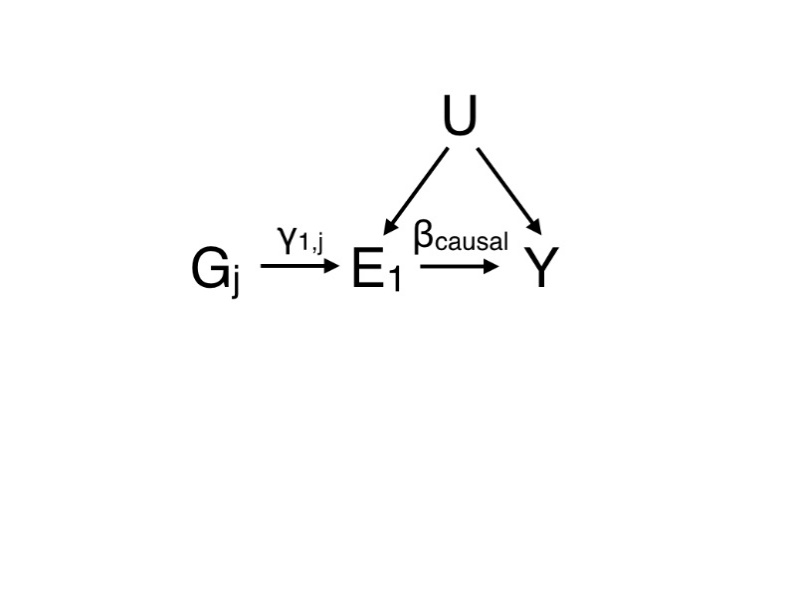


Stage 1: Regress E_1_ on G_j_

$E_{1,i}=\gamma_{0}+\gamma_{1,j}G_{j,i}+\varepsilon_{E_{1},i}$

Stage 2: Regress Y on $\hat{E}_{1,i}$ (fitted value of $E_{1,i}$ from stage 1)

$$Y_{i}=\Gamma_{0}+{\beta_{causal}\hat{E}}_{1,i}+\varepsilon_{Y,i}$$

**Supplementary Tables**

**Table S1.** Multivariate^a^ linear regression to examine the association of telomere length (dependent variable) with demographic, clinical, and METH use variables (independent variables) for METH users with and without a history of polysubstance use

| Independent variable | | Dependent variable: Square root of the relative T/S ratio | | | |
| --- | --- | --- | --- | --- | --- |
|  |  | With no history of polysubstance use  (N=119) | | With a history of polysubstance use  (N=68) | |
|  |  | Beta | 95% CI | Beta | 95% CI |
| Age | | -0.002 | -0.045, 0.041 | -0.021 | -0.064, 0.021 |
| Sex | |  |  |  |  |
| Female | | -0.011 | -0.356, 0.333 | -0.224 | -0.542, 0.094 |
| Marital status | |  |  |  |  |
| Married | | 0.116 | -0.166, 0.399 | 0.296 | -0.033, 0.624 |
| Education years | | -0.002 | -0.064. 0.061 | -0.0001 | -0.061, 0.061 |
| Smoking | |  |  |  |  |
| Cumulative smoking dose (pack-years) | | **0.015** | **0.004, 0.025** | -0.008 | -0.015, 0.0004 |
| ACEs score^b^ | | -0.009 | -0.067, 0.052 | -0.008 | -0.056, 0.041 |
| METH use variable | |  |  |  |  |
| Onset age of METH use | -0.008 | -0.031, 0.013 | -0.006 | -0.029, 0.018 |  |
| Duration of METH use | **-0.004** | **-0.007, -0.00003** | -0.001 | -0.003, 0.001 |  |
| Maximum frequency of METH use | 0.002 | -0.003, 0.006 | -0.001 | -0.002, 0.001 |  |

|  |  |  |  |  |
| --- | --- | --- | --- | --- |

^a^ Multivariate analysis adjusted for age, sex, marital status, educational attainment, total ACEs score, cumulative smoking dose, and METH use variables

^b^ ACE score: the summed number of ACEs categories for each participant (range, 0-9)

Bold font indicates statistical significance (p < 0.05)

Abbreviations: 95% CI: 95% confidence interval.

**Table S2.** Multivariate^a^ linear regression to examine the association of telomere length (dependent variable) with demographic, clinical, and METH use variables (independent variables) for METH users with positive and negative urine METH test results

| Independent variable | | Dependent variable: Square root of the relative T/S ratio | | | |
| --- | --- | --- | --- | --- | --- |
|  |  | Urine positive for METH  (N=70) | | Urine negative for METH  (N=117) | |
|  |  | Beta | 95% CI | Beta | 95% CI |
| Age | | 0.007 | -0.043, 0.057 | -0.009 | -0.054, 0.036 |
| Sex | |  |  |  |  |
| Female | | -0.012 | -0.323, 0.300 | -0.251 | -0.606, 0.103 |
| Marital status | |  |  |  |  |
| Married | | 0.077 | -0.191, 0.344 | 0.564 | 0.177, 0.950 |
| Education years | | 0.003 | -0.060. 0.065 | 0.010 | -0.053, 0.072 |
| Smoking | |  |  |  |  |
| Cumulative smoking dose (pack-years) | | 0.003 | -0.008, 0.015 | 0.002 | -0.007, 0.011 |
| ACEs score^b^ | | -0.002 | -0.047, 0.043 | -0.032 | -0.106, 0.043 |
| METH use variable | |  |  |  |  |
| Onset age of METH use | -0.002 | -0.024, 0.020 | -0.013 | -0.037, 0.011 |  |
| Duration of METH use | **-0.003** | **-0.005, -0.001** | -0.002 | -0.004, 0.001 |  |
| Maximum frequency of METH use | -0.001 | -0.003, 0.001 | -0.0002 | -0.003, 0.002 |  |

| Polysubstance use | **0.251** | **0.049, 0.452** | 0.020 | -0.025, 0.288 |
| --- | --- | --- | --- | --- |

^a^ Multivariate analysis adjusted for age, sex, marital status, educational attainment, total ACEs score, cumulative smoking dose, polysubstance use, and METH use variables

^b^ ACE score: the summed number of ACEs categories for each participant (range, 0-9)

Bold font indicates statistical significance (p < 0.05)

Abbreviations: 95% CI: 95% confidence interval.

**Supplementary Figure S1. Square root transformation to normalize the LTL measures.**

Histograms demonstrating the distributions of LTL measures (i.e., the relative t/s ratio) before transformation in (a) all participants, (b) METH users, and (c) non-users; and the distributions of square-root transformed LTL measures in (d) all participants, (e) METH users, and (f) non-users.


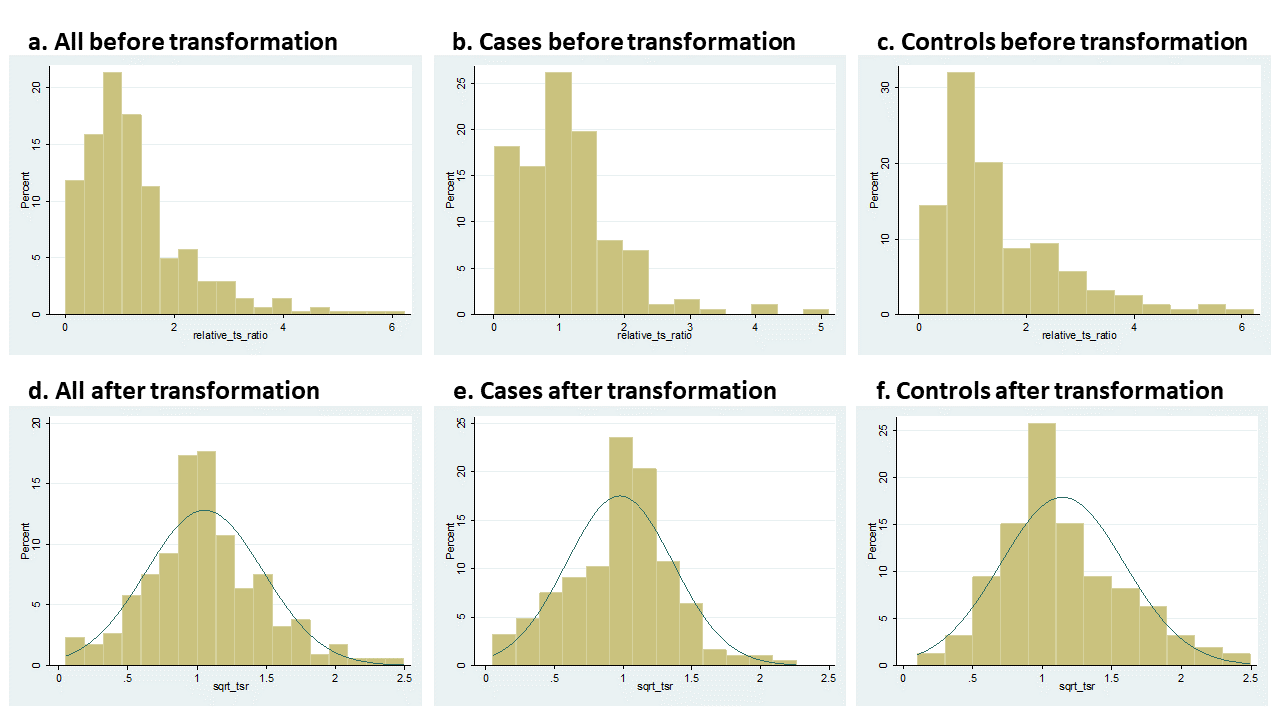


**Supplementary Figure S2.**

Genome-wide association of duration of METH use graphed by chromosome positions and −log10 P-value (Manhattan plot), and (b) quantile-quantile plot of 5,374,651 imputed and genotyped SNPs.

1. Manhattan plot: The P-value for each SNP was obtained from linear regression association test using Plink. The Y axis shows −log10 P-values, and the X axis shows chromosome positions. Horizontal red line represents the thresholds of P=5E-08 genome-wide significance. We identified one SNP rs6585206 genome-wide significantly associated with METH use duration (P=8.76E-13).
2. QQ-plot: The Y axis shows observed −log10 P-values, and the X axis shows the expected −log10 P-values. Each SNP is plotted as a dot, and the red line indicates null hypothesis of no true association. Deviation from the expected P-value distribution is evident only in the tail area, with a lambda GC of 1.08, suggesting that population stratification was adequately controlled.


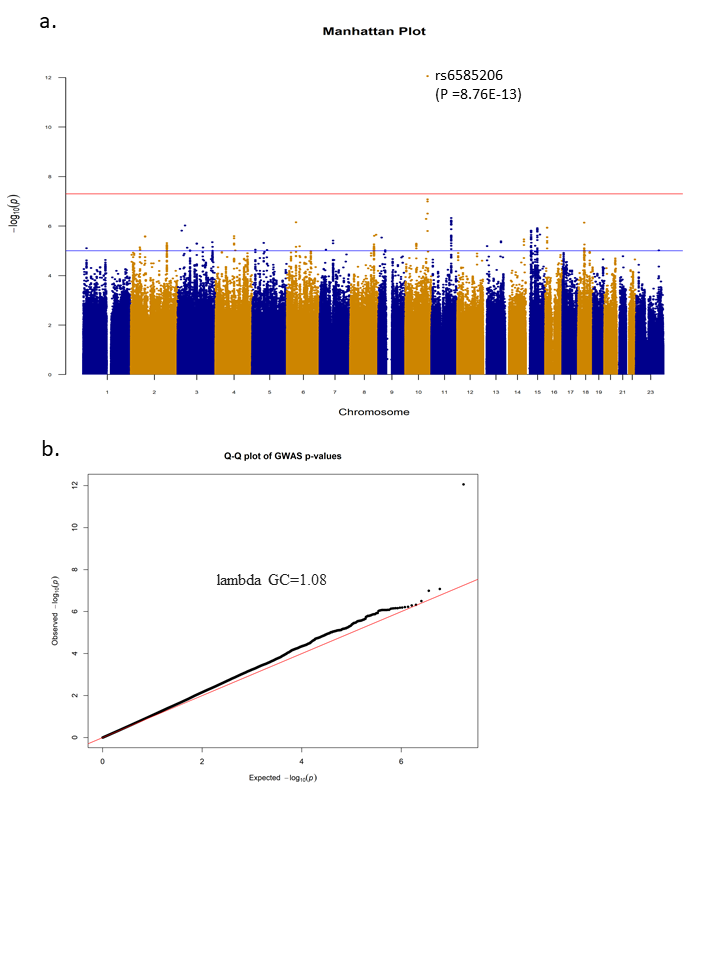

Supplement: Supplementary file 1 — Supplementary Methods and Supplementary Tables [file 41398_2021_1640_MOESM1_ESM.docx]
